# Annual rhythms in adults’ lifestyle and health (ARIA): protocol for a 12-month longitudinal study examining temporal patterns in weight, activity, diet, and wellbeing in Australian adults

**DOI:** 10.1186/s12889-020-10054-3

**Published:** 2021-01-07

**Authors:** Rachel G. Curtis, Timothy Olds, François Fraysse, Dorothea Dumuid, Gilly A. Hendrie, Adrian Esterman, Wendy J. Brown, Ty Ferguson, Rajini Lagiseti, Carol A. Maher

**Affiliations:** 1 Alliance for Research in Exercise, Nutrition and Activity, UniSA Allied Health and Human Performance, University of South Australia, Adelaide, SA Australia; 2 Health and Biosecurity, Commonwealth Scientific and Industrial Research Organisation, Adelaide, SA Australia; 3 UniSA Clinical and Health Sciences, University of South Australia, Adelaide, SA Australia; 4 School of Human Movement and Nutrition Sciences, University of Queensland, Brisbane, QLD Australia

**Keywords:** Physical activity, Sedentary behaviour, Sleep, Diet, Weight, Overweight, Obesity, Epidemiology, Compositional data

## Abstract

**Background:**

Almost one in three Australian adults are now obese, and the rate continues to rise. The causes of obesity are multifaceted and include environmental, cultural and lifestyle factors. Emerging evidence suggests there may be temporal patterns in weight gain related, for example, to season and major festivals such as Christmas, potentially due to changes in diet, daily activity patterns or both. The aim of this study is to track the annual rhythm in body weight, 24 h activity patterns, dietary patterns, and wellbeing in a cohort of Australian adults. In addition, through data linkage with a concurrent children’s cohort study, we aim to examine whether changes in children’s body mass index, activity and diet are related to those of their parents.

**Methods:**

A community-based sample of 375 parents aged 18 to 65 years old, residing in or near Adelaide, Australia, and who have access to a Bluetooth-enabled mobile device or a computer and home internet, will be recruited. Across a full year, daily activities (minutes of moderate to vigorous physical activity, light physical activity, sedentary behaviour and sleep) will be measured using wrist-worn accelerometry (Fitbit Charge 3). Body weight will be measured daily using Fitbit wifi scales. Self-reported dietary intake (Dietary Questionnaire for Epidemiological Studies V3.2), and psychological wellbeing (WHOQOL-BREF and DASS-21) will be assessed eight times throughout the 12-month period. Annual patterns in weight will be examined using Lowess curves. Associations between changes in weight and changes in activity and diet compositions will be examined using repeated measures multi-level models. The associations between parent’s and children’s weight, activity and diet will be investigated using multi-level models.

**Discussion:**

Temporal factors, such as day type (weekday or weekend day), cultural celebrations and season, may play a key role in weight gain. The aim is to identify critical opportunities for intervention to assist the prevention of weight gain. Family-based interventions may be an important intervention strategy.

**Trial registration:**

Australia New Zealand Clinical Trials Registry, identifier ACTRN12619001430123. Prospectively registered on 16 October 2019.

## Background

Australian obesity rates are amongst the highest in the world. Almost one in three Australian adults are obese (31%), and a further third are overweight (36%) [1]. Out of the 34 OECD countries, Australia’s adult obesity rate ranks 5th highest, and has shown large increases over the past 10 years, in contrast to counties that are reporting modest growth (Canada and Spain) or even stabilisation (US and UK) [2]. Excess weight, and obesity in particular, is a major risk factor for numerous health conditions including cardiovascular disease, type 2 diabetes, some musculoskeletal conditions and some cancers [3–5]. In 2017, the total annual cost of obesity in Australia was estimated at $11.8 billion [6]. Fully understanding the mechanisms underpinning obesity is crucial if targeted and effective obesity programs are to be developed.

The determinants of obesity are complex. Ecological models recognise they include individual, social, and environmental factors. In particular, excess energy intake through unhealthy eating patterns, insufficient energy expenditure due to lack of physical activity and excess sedentary behaviour, and unhealthy sleep, have been highlighted as key modifiable individual risk factors [7, 8]. Such risk factors are socially and geographically patterned and tend to cluster within individuals and families. In particular, people with lower socioeconomic status are more likely to be overweight or obese, this gap is wider in women, and people in regional areas are more likely to be overweight or obese [9]. Family factors, such as meal-time patterns, parenting style and work patterns have also been show to influence childhood obesity [10]. While we recognise that these determinants have genetic, social and geographical origins, less attention has been paid to temporal patterning.

### Weight gain across the year

Longitudinal studies suggest that, on average, Australian adults aged less than 65 years gain around 0.5 kg [11] and 0.6 cm of waist girth [11, 12] per year. However, the temporal patterning of this weight gain across the year is poorly understood. Research suggests that both weekly and annual cycles in day type (weekday and weekend days), cultural events, and season intertwine to influence adults’ weight gain, activity patterns and eating patterns across the year, as outlined below.

Studies of days of the week suggest health behaviours may differ between weekdays and weekend days. For example, a US study of 9000 adults found that energy intake was 6% higher on weekend days compared to weekdays, with increased proportions of energy from fat and alcohol on weekends [13]. In addition, studies have suggested physical activity is lower on weekend days than weekdays [14, 15], while sleep duration is longer on weekend days [16, 17].

Studies of festive periods, and particularly the “holiday season” (in the West, from around late November through to early January, taking in Thanksgiving, Christmas and New Year’s Eve) show that weight gain coincides with cultural celebrations. US “holiday season” weight gain is consistently 0.4–0.9 kg [18], and it appears that average weight gain over this 6-week period may account for the majority of annual weight gain [19]. A study of weight change in three Northern Hemisphere countries (US, Germany and Japan) found that weight gain coincided with cultural celebrations, however the relative importance of celebrations for weight gain varied between countries – in particular, the percentage weight gain at Christmas and Easter in Germany was almost double that for the US, and in Japan, most weight gain occurred during the May “Golden Week” celebration [20].

Studies of seasonal changes in weight and health behaviour suggest weight peaks in winter and troughs in summer [21]. Seasonal differences in energy and nutrient intake have been reported, though they are generally small in magnitude, and the direction of such differences is inconsistent between studies [13, 22]. Similarly, some studies have shown that adults sleep more in winter [23, 24], while others have shown no seasonal difference in sleep [25, 26]. Adult studies in the US and Europe found that people are relatively more active in summer than in winter [27–29]. However, a recent study of children suggests different patterns may occur in Australia and Canada– while Canadian children were relatively more active in summer than winter, the opposite was seen for Australian children [30]. The authors suggested that this may be a product of Australia’s winters being relatively mild compared with Canada’s, and Australia’s summers being relatively harsher.

In sum, the evidence suggests that temporal patterning in activity and diet across the year leads to cyclical spikes in weight gain, which accrue over time. However, research to date has been piecemeal, focussed on only diet, or activity patterns or weight gain, and not all three simultaneously. Additionally, Australian data are lacking; it is possible that international findings may not reflect Australian patterns, due to key differences in timing of festivities by season (e.g. Australian Christmas falls in summer) and climate (e.g. Australian winters are relatively mild and summers relatively harsh). Finally, most research on the effects of daily physical activity, sedentary behaviour and sleep has examined the activities in isolation and applied standard multivariate statistical techniques which assume that daily activities are independent. However, activity data are naturally co-dependent – they occur in a finite 24-h window, thus if one activity increases (e.g. moderate to vigorous physical activity), then another must decrease (e.g. sleep). Research employing frequent waves of data collection, taking into account the compositional nature of activity data, is required to understand temporal patterns of weight gain, activity and dietary intake in order to enable the design and implementation of interventions at the time they are needed most. For example, if most annual weight gain occurs at Christmas, and this coincides with unhealthy eating patterns, dietary interventions could be targeted in the lead up to Christmas. This aims of this study are therefore to:

1. Describe the annual rhythms in adults’ body weight, daily activities and eating patterns, and determine whether changes in body weight across the year are associated with changes in activity and diet compositions.
2. Determine whether changes in children’s body mass, activity and diet compositions are related to those of their parents, and
3. Examine whether parental factors (parenting style and home environment) are associated with changes in children’s body mass, activity and diet compositions.

## Methods

### Study design

This is a prospective cohort study “Annual Rhythms In Adults’ lifestyle and health” (ARIA) that will measure weight, daily activity, dietary intake, and wellbeing over a 12 month period. The study has been approved by the University of South Australia Human Research Ethics committee (Protocol number: 201901) and registered on the Australian New Zealand Clinical Trial Registry (Trial ID: ACTRN12619001430123). The South Australian Department for Education and Catholic Education South Australia has provided approval to match ARIA data with data from children in a concurrent cohort study, the “Life on Holidays” study [31], as further described below.

### Participants and procedure

A community-based sample of 375 predominantly middle-aged adults will be recruited from the greater metropolitan area of Adelaide, South Australia, using two methods. Firstly, all parents and guardians of children enrolled in a separate three-year cohort study, “Life on Holidays” [31], will be invited to participate via email or postal invitation. In the Life on Holidays study, 380 children were recruited from Adelaide metropolitan schools in two waves commencing Feb 2019 and Feb 2020. Schools were randomly selected and invited from low, middle and high socio-educational advantage tertiles to ensure a representative sample. Anticipating the uptake from these parents to be a challenge, given the burden of participating in simultaneous 12-month trials, a second recruitment method is planned in case it is needed. Parents meeting the inclusion criteria will also be recruited through general advertising (social media posts, paid Facebook advertisements, print media). Mirroring the Life on Holidays protocol, participants will be enrolled in two waves (December 2019 and December 2020). Inclusion criteria are parent/guardian of a child enrolled in Life on Holidays or parent/guardian of child aged 5 to 12 years, 18 to 65 years old, residing in greater metropolitan Adelaide, with access to a Bluetooth-enabled mobile device or computer and home internet, proficiency in English, and ambulant. Exclusion criteria are pregnancy, having an implanted electronic medical device, or experiencing or receiving treatment for any life-threatening condition which impacts daily lifestyle and health.

Participants will comprise two cohorts: those beginning in December 2019 and those beginning in December 2020. A baseline face-to-face home visit will be conducted between August and November in the year of commencement, where participants will have their height measured and be given a Fitbit Charge 3 activity monitor and Fitbit Aria body weight scale (Aria 2 or Aria Air scale; Fitbit Inc., San Francisco, CA, USA). Participants will be asked to wear the activity monitor continuously except during water-based activities, and to weigh themselves daily. To enable remote collection of daily activity and weight data, participants will authorise bespoke software, “Fitnesslink”, to access their Fitbit user account data, including user profile details, sleep, activity and weight data, and device data (battery status and time of most recent sync).

**Fig. 1:**
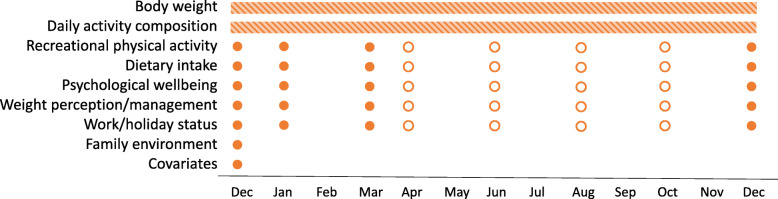
ARIA assessment schedule. Bars indicate continuous assessment. Full dots indicate assessments coinciding with Life on Holidays assessments. Empty dots indicate assessments at additional timepoints

Participants will complete a baseline survey about their demographic, health, and lifestyle characteristics either before or during the home visit. They will also complete eight online surveys about recreational physical activity, dietary intake, psychological wellbeing, weight perception/management, and work/holiday status over 12 months (see Fig. 1). Four of the online surveys are scheduled to coincide with assessments conducted in the Life on Holidays study to allow for within-family comparisons.

Upon completion of the study, participants will be provided an honorarium of $100 and will be allowed to keep the Fitbit Charge 3 and weight scales.

### Measures

#### Body weight

Body weight will be measured using Fitbit Aria electronic weight scales (Fitbit Inc., San Francisco, CA, USA) [32, 33]. Participants will be encouraged to weigh themselves preferably daily but at least weekly, in the morning, whilst wearing minimal clothing, prior to meals and after voiding. Participants who do not wish to weigh in the morning will be asked to weigh at the same time on each occasion. Body weight data will be uploaded to participants’ Fitbit accounts and collected remotely via the Fitnesslink software. Height will be measured at baseline home visit using a stadiometer (Leicester Height Measure MKII). Measures will be conducted according to International Society for the Advancement of Kinanthropometry assessment procedures [34].

#### Daily activity composition

Daily activity composition will be measured continuously using Fitbit Charge 3 activity monitors (Fitbit Inc., San Francisco, CA, USA). Participants will be asked to wear the device on their non-dominant wrist 24-h a day, except during water activities and device charging, and to sync data to their Fitbit account at least every 5 days. Data will be collected remotely via the Fitnesslink software, created especially for this study. Each minute in every 24-h period will be classified as sleep, sedentary, light, moderate or vigorous physical activity according to Fitbit’s proprietary algorithm, which also provides bedtime and rise time. Minutes classified as sedentary (recorded when no other activities are detected) AND with missing heart rate data will be classified as non-wear. Fitbit activity monitors are commonly used in biomedical research [35, 36] and have acceptable validity for moderate-to-vigorous physical activity (Fitbit Flex compared to Actigraph GT3X+ *r =* 0.731 [37]), sleep (Fitbit Charge 2 compared to polysomnography sensitivity = 0.96, specificity = 0.61 [38]; Fitbit Flex compared to polysomnography *r =* 0.97 [39]) and total daily energy expenditure (Fitbit Flex compared to doubly labelled water in free-living conditions r_s_ = 0.84 [40]).

#### Recreational activity.

Recreational physical activity will be assessed at each timepoint using items from the How Areas in Brisbane Influence Health and Activity (HABITAT) study [41] which will be modified to reflect recreational activity over the previous month. Participants rate how often they did 15 activities (e.g. running or jogging, team sports, water activities) on a 5-point scale (never, once a month, once every 2 weeks, once a week, more than once a week). Participants will also report the number of hours they watch television or use an electronic device (computer, tablet, smartphone, video games) in their free time (1) on typical a weekday and (2) on typical a weekend day.

#### Dietary intake

Dietary intake will be assessed at each timepoint using the online Dietary Questionnaire for Epidemiological Studies (DQES v3.2; Cancer Council Victoria) [42], which will be modified to reflect diet over the previous month. The DQES v3.2 is an online self-administered questionnaire that estimates nutritional intake (grams of foods, macro nutrients and micronutrients) based on 144 foods and beverages using nutritional information from the NUTTAB 2010 [43] and AUSNUT 2007 [44]. The DQES has been demonstrated to have good reproducibility and has good agreement with weighed food records [45, 46].

#### Psychological wellbeing

Quality of life will be assessed at each timepoint using the World Health Organization Quality of Life assessment 26-item version (WHOQOL-BREF) [47]. WHOQOL-BREF is a self-report questionnaire which measures four broad domains: physical health, psychological health, social relationships and environment. The WHOQOL-BREF has good discriminant validity, content validity, test-retest reliability and internal consistency [47–49]. The online version of the questionnaire is a valid and reliable alternative to the original paper-based version of the questionnaire [50].

Symptoms of depression, anxiety and stress will be assessed at each timepoint using the 21-item short-form Depression Anxiety Stress Scale (DASS-21) [51]. Participants rate the extent to which they agree with 21 statements such as “I found it hard to wind down” on a 5-point scale from 1 “Did not apply to me at all” to 5 “Applied to me very much, or most of the time”. The DASS-21 has good convergent and discriminant validity when compared with other validated depression and anxiety measures, adequate construct validity, and high reliability [51–53].

#### Weight perception and management

At baseline, participants will report whether they consider themselves to be an acceptable weight, underweight or overweight and whether they have been weight stable (within 5% of body weight) in the past 3 months [54]. At each timepoint, participants will report whether they have used a range of weight control practices in the past month (adapted from the Behavioral Risk Factor Surveillance System [55]) and whether they currently use any medication from a list of six broad categories that can be associated with weight change [56, 57].

#### Work/holiday status

At baseline, participants will report their occupation (open-ended response), whether they work shift work that includes some night shifts, and whether they regularly work on weekends. At each time point, participants will report their average hours of work outside the home (none, less than 15 h per week, 15 to 30 h per week, fulltime 36h hours per week), whether (and on what days) they have been on holidays or had annual leave since the last assessment (in the previous month at baseline), and whether (and on what days) they were away from home during that time.

#### Family environment

A range of family environment measures will be completed at baseline. Parenting style will be measured using the parenting warmth and parenting consistency scales from the Longitudinal Study of Australian Children [58]. Participants will report how they encourage their child’s physical activity on a 4-item scale adapted from adapted from ISCOLE Neighborhood and Home Environment Questionnaire [59] and describe how meals take place on the “Structure of Family Meals” subscale of the Meals in Our Household Questionnaire [60]. Participants will also report whether a TV, computer or video game system (non-hand held; PlayStation, Xbox, etc.) are in their bedroom and their child’s bedroom (adapted from ISCOLE [59]).

#### Baseline covariates

At baseline, participants will report their date of birth, sex, country of birth, marital status (never married, widowed, divorced, separated but not divorced, married or de facto), number of adults and number of children at home. Socioeconomic status measures include highest education level (below year 10, year 10, year 11, year 12 or equivalent, certificate III/IV, advanced diploma/diploma, bachelor degree, postgraduate or higher degree), combined gross household income (<$50,000, $50,000–$99,999, $100,000–$199,999, ≥$200,000), number of motorised vehicles in the household, and type of television/streaming service. Participants will report their smoking status and whether they have any of ten listed chronic conditions (adapted from the Australian National Health Survey [1]). Participants will also report their sleep chronotype (single item from both the Horne-Östberg Morningness-Eveningness-Questionnaire [61]) and complete an 8-item scale assessing their tendency towards routine [62].

### Power calculation

Due to the complex nature of the multilevel model addressing Aim 1, current sample size software is unable to estimate the required sample size. We have therefore calculated the required sample size for a multivariable linear regression model, using conservative estimates, with added adjustments for clustering within families. For the initial estimate, a sample size of 226 achieves 90% power to detect an r^2^ of 0.10 attributed to 10 independent variables when the significance level (alpha) is 0.025. In addition, data are clustered within families, with an expected average cluster size of 1.5, and an ICC of 0.7. This equates to a design effect of 1.35, bringing the required total to 305 subjects. Allowing for 20% withdrawals/loss to follow up, the final sample size is 371. These estimates are approximate, but suggest that the anticipated sample size of 375 will provide sufficient power.

### Statistical analysis

Longitudinal compositional data analysis methods will be used to assess activity and diet compositions. To account for the inherent co-dependency of compositional parts (proportions of time or macronutrients), compositions will be expressed as sets of isometric log ratios (ilrs) before their inclusion in statistical models [63, 64]. Aim 1 will use data from all participants. Aims 2 and 3 will use data only from parents/guardian of children enrolled in Life on Holidays and matched data from children in Life on Holidays.

#### Aim 1

Annual rhythms will be described graphically using Loess curves. Associations between change in weight (dependent variable) and changes in activity and diet compositions (independent variables) will be examined using multilevel modelling to adjust for non-independence of data, allowing for nesting of repeated measures within individuals and individuals within families. Diet and activity will be examined simultaneously to identify unique effects on weight. Covariates will include age, sex, socioeconomic status, and total energy intake.

#### Aim 2

Associations between change in children’s (dependant variables) and parents’ (independent variables) weight, activity, and diet compositions will be examined using a series of multilevel models to adjust for non-independence of data. Covariates will include parental and child age, sex, socioeconomic status, and child total energy intake.

#### Aim 3

Factor analysis will be used to derive factors from the baseline family environment items. The association between change in children’s body mass index z-score, and activity and diet compositions (dependant variables) and family environment (independent variable) will be examined using a series of multilevel models. Covariates will include parental and child age, sex, socioeconomic status, and total energy intake.

## Discussion

A main strength of this study is its novelty — it will be the first in the world to simultaneously examine temporal patterns in weight, daily activities and diet. Additional strengths are the use of wireless tracker technology and wifi scales to provide high fidelity information about adults’ activity patterns and weight changes across the full year, with low participant burden. The study will use a compositional analysis approach, which is quickly gaining traction in time-use epidemiology. In addition, the opportunity to link parents’ and children’s data will allow examination of the interplay between temporal and family determinants of obesity.

Potential limitations must also be acknowledged. The study only targets adults who are parents. While the majority of Australian adults are parents (approximately 75% of 45–49 year old Australian women have children [65]), we acknowledge that their data may not represent adults who are not parents, or likewise those who are not motivated to participate in a research study. In addition, participants are recruited from a single Australian city (Adelaide, population 1.3 million); however, surrounding areas are included so that participants are drawn from metropolitan, suburban areas, and rural areas. It is possible that the weighing regimen may cause reactivity, given that frequent self-weighing can be an effective weight intervention strategy [66]. Reactivity will be reduced by allowing weekly weighing (i.e. not enforcing daily weighing), explaining to participants that the purpose of the study is to monitor usual lifestyle (not intervene), and through use of automated data collection (preventing the need for journaling).

With nearly one-third of Australian adults obese, a further one-third overweight, and rates continuing to rise, obesity is one of the greatest health challenges facing Australia. To date, public health obesity campaigns have typically intervened by geography (e.g. rural and remote), demography (e.g. targeted ethnic groups) or socioeconomically (e.g. low SES families). However, it appears likely that temporal factors, such as day type, cultural celebrations and season, may also play a key role in weight gain. It is hoped that this study’s findings will reveal critical moments for intervention to assist the prevention of weight gain.

Furthermore, the study will examine the interplay between temporal and family determinants of obesity. If our results reveal intergenerational concordance in temporal patterns of weight gain, activity and diet compositions, this would suggest that carefully-timed, family-based interventions could be an important intervention strategy. Recent research suggests that parent-child obesity interventions may be a particularly effective [67], yet under-used, intervention strategy.

## Data Availability

N/A

## Abbreviations

DASS-21: Depression Anxiety Stress Scale
DQES: Dietary Questionnaire for Epidemiological Studies
Ilrs: Isometric log ratios
WHOQOL-BREF: World Health Organization Quality of Life assessment 26-item version
